# Coronavirus disease 2019 and pityriasis rosea: A review of the immunological connection

**DOI:** 10.1111/1346-8138.16482

**Published:** 2022-06-08

**Authors:** Francesco Borgia, Federica Li Pomi, Clara Alessandrello, Mario Vaccaro, Giovanni Pioggia, Sebastiano Gangemi

**Affiliations:** ^1^ Section Of Dermatology, Department of Clinical and Experimental Medicine University of Messina Messina Italy; ^2^ School and Operative Unit of Allergy and Clinical Immunology University of Messina Messina Italy; ^3^ Institute for Biomedical Research and Innovation (IRIB) National Research Council of Italy (CNR) Messina Italy

**Keywords:** cytokine storm, interferon, immune system, pandemic respiratory infection, pityriasis rosea, severe acute respiratory syndrome coronavirus 2, T cells, telemedicine, T‐helper 17, viral reactivation

## Abstract

Severe acute respiratory syndrome coronavirus 2 (SARS‐CoV‐2) is characterized by the activation of a cytokine storm derived from an excess release of cytokine (interleukin [IL]‐6, interferon [IFN] I, C‐X‐C motif chemokine ligand [CXCL]10, tumor necrosis factor [TNF]‐α, macrophage inflammatory protein [MIP]1) due to an uncontrolled immune activation. There has been a fivefold increase in the number of cases of pityriasis rosea during the SARS‐CoV‐2 pandemic. Using the keywords “pityriasis” and “COVID‐19”, we carried out a PubMed search, including all articles in the English language published until November 2021. We aimed to investigate the possible connection between SARS‐CoV‐2 and pityriasis rosea (PR). Pityriasis could be considered an immunological disease due to the involvement of cytokines and chemokines. Our analysis yielded 65 articles of which 53 were not considered; the others (*n* = 12) concerning the association between PR and COVID‐19 were included in our study. We suggest two mechanisms underlying the involvement of the skin in viral infections: (i) viruses directly affecting the skin and/or inducing host immune response thus causing cutaneous manifestations; and (ii) viruses as a possible inducer of the reactivation of another virus. The first mechanism is probably related to a release of pro‐inflammatory cytokine and infection‐related biomarkers; in the second, several pathways could be involved in the reactivation of other latent viruses (human herpesviruses 6 and 7), such as a cytokine–cytokine receptor interaction, the Janus kinase–signal transducer and activator of transcription signaling pathway, and the IL‐17 signaling pathway. We thus believe that a cytokine storm could be directly or indirectly responsible for a cutaneous manifestation. More investigations are needed to find specific pathways involved and thus confirm our speculations.

## 1 INTRODUCTION

Since its initial outbreak in China’s Hubei province in December 2019, severe acute respiratory syndrome coronavirus 2 (SARS‐CoV‐2) and the resulting pandemic, coronavirus disease 2019 (COVID‐19) has caused drastic consequences, affecting every aspect of personal and work life. As of December 2021, the World Health Organization (WHO) estimated over 271 million confirmed COVID‐19 cases, including over 5 million deaths.^1^

### 1.1 COVID‐19: Immunological aspects

Severe acute respiratory syndrome coronavirus 2 infection is characterized by rapid virus replication. In severe cases, a cytokine storm, derived from an uncontrolled overproduction of soluble markers of inflammation, may result in edema, congestion of the lung, thickening of interstitial tissue, and augmented exudation in the alveolar space. Patients affected by SARS‐CoV‐2 show a reduction in both T helper (Th) and cytotoxic T lymphocytes (CTL) (with the Th/T‐suppressor ratio within the normal limits). Critical patients also have fewer regulatory T cells, both naive and induced Treg.

The level of CD8^+^ T cells is reduced in infected patients and negatively correlates with the C‐reactive protein (CRP), the erythrocyte sedimentation rate (ESR), and interleukin (IL)‐6. On the other hand, the CD4^+^/CD8^+^ ratio positively correlates with CRP, ESR, and IL‐6: the higher the viral load, the lower the number of CD4^+^ and CD8^+^.

Even blood concentrations of IL‐2R and IL‐6 seem to be positively correlated with disease severity. High levels of IL‐6 are considered a negative prognostic factor in patients with SARS‐CoV‐2. IL‐6 inhibits CD8^+^ T cells, thus reducing interferon (IFN)‐γ production and blocking specific cytokine signaling as suppressor of cytokine signaling 3 (SOCS‐3) and the consequent cell‐mediated antiviral response during a cytokine storm.^2^

Several research papers seem to confirm the role of the mediators involved in the Th17 response, such as IL‐17, IL‐21, IL‐22, and granulocyte‐macrophage colony‐stimulating factor (GM‐CSF). All these mediators perform pro‐inflammatory actions by increasing inflammatory cytokines (IL‐1β, IL‐6, tumor necrosis factor [TNF]‐α, and granulocyte colony‐stimulating factor [G‐CSF]) and chemokines (IFN‐γ‐induced protein 10 [IP‐10], IL‐8, and macrophage inflammatory protein [MIP]2A), which recruit more immune cells responsible for tissue injury and more severe Sars‐CoV‐2 damage.^2^ Khesht et al. speculate that the increase of TNF‐α and IL‐6 might reduce the suppressive function of Treg by inhibiting the transforming growth factor (TGF)‐β and forkhead box P3 (FOXP3).^3^

In mild cases, an anti‐inflammatory response mediated by IL‐4 and IL‐10 is prevalent, which suppresses Th1 cell differentiation and proliferation and inhibits pro‐inflammatory cytokine production.^2^

**FIGURE 1:**
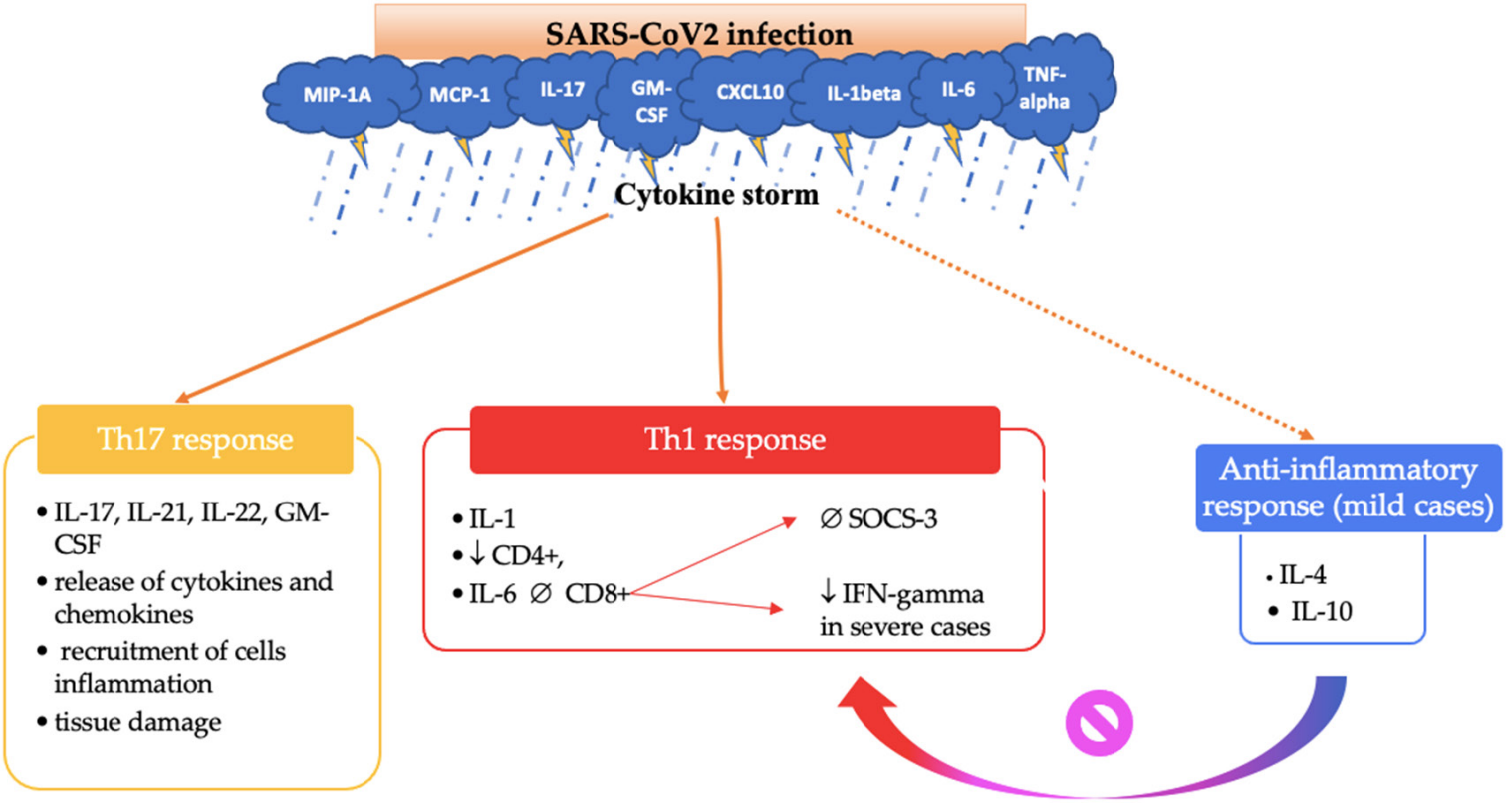
The immunological mechanisms activated by SARS‐CoV2 infection are closely related to the cytokine storm and they can be divided into three branches: Th1‐mediated response, Th17‐mediated response, and anti‐inflammatory response which is generally activated in cases of mild disease and could inhibit the Th1 response. CXCL10, C‐X‐C motif chemokine ligand; GM‐CSF, granulocyte‐macrophage Colony‐Stimulating Factor; IFN‐gamma, Interferon‐gamma; IL‐1,4,6,10,21,22, Interleukin‐1,2,4,6,10,21,22; MCP‐1, monocyte chemoattractant protein 1; MIP‐1A, macrophage inflammatory protein‐1 alpha; SOCS‐3, Suppressor of cytokine signaling 3; TNF, tumor necrosis factor.

Increasing evidence suggests that type I IFN pathways are key to the immune‐mediated clearance of SARS‐CoV‐2. Severe COVID‐19 infections have been correlated with low blood levels of type I IFN and low white blood cell expression of type I IFN‐inducible genes. In this way, deficiencies in the type I IFN pathway, due to inherited mutations or the development of autoantibodies, predispose patients to develop severe COVID‐19^4^ Below, Figure 1 summarizes the main immunological mechanisms through which SARS‐CoV‐2 causes clinical manifestations.

### 1.2 Cutaneous manifestations of SARS‐CoV‐2

Since the start of the COVID‐19 pandemic, multiple studies have reported that SARS‐CoV‐2 can be associated with dermatological manifestations. The association between different types of cutaneous involvement and the severity of COVID‐19 is likely due to the varying immune response following SARS‐CoV‐2 infection. Indeed, livedo racemosa and retiform purpura are associated with a more severe disease course and higher mortality. In contrast, viral exanthem and inflammatory lesions, such as urticarial and vesicular eruptions, seem to be associated with a less severe COVID‐19 disease course which were reported more frequently for inpatients.^5^ In some cases, cutaneous involvement may represent a visual marker for the early identification of infection. COVID‐19 cutaneous clinical patterns include morbilliform, vesiculopapular, pernio‐like lesions and purpura.^6^ SARS‐CoV‐2 is related to the onset of several viroses, such as those mediated by herpes simplex virus (HSV), varicella zoster virus (VZV), cytomegalovirus (CMV), and Epstein–Barr virus (EBV), which can appear with a plethora of skin manifestations, through a possible viral reactivation mechanism.^7^ COVID‐19 can be also associated with various dermatological diseases. Among these, a relative increase in pityriasis rosea (PR) and PR‐like rash was reported during the pandemic, despite restrictions imposed by social distancing and despite the recommendation to limit non‐urgent outpatient services during the pandemic.^8^, ^9^ Using clinical and histopathological findings, Öncü et al. managed to establish the diagnosis of PR after COVID‐19 infection. Histopathology of the biopsy of one lesion showed focal parakeratotic peaks, spongiosis, focal spongiotic vesiculation, lymphocyte exocytosis, mildly irregular acanthosis with mild homogenization of collagen in the dermis, mild to moderate perivascular erythrocyte infiltration in the superficial vascular plexus, scattered lymphocyte infiltration, and sparse lymphocytes.^10^

### 1.3 PR: Pathogenesis and immunological aspects

Pityriasis rosea is an acute, self‐limiting exanthematous disease, predominantly affecting children and young adults. Some epidemiological features (seasonal variation and clustering in households) suggest that PR may be infectious. Reactivation of latent human herpesviruses (HHV)‐6 and ‐7 infections has been suggested as the most probable etiological agent.

Pityriasis rosea typically begins with a single, erythematous scaly plaque (herald patch or mother spot) followed by a secondary eruption consisting of smaller scaly papulosquamous lesions on the cleavage lines of the trunk in a Christmas tree‐like shape. It appears in crops at intervals of a few days and reaches its acme in approximately 2 weeks. The duration may vary from 2 weeks to a few months, and constitutional symptoms may precede or accompany the skin eruption (general malaise, sore throat, mild fever, fatigue, nausea, headache, joint pain, swelling of lymph nodes).^11^, ^12^

The etiopathogenetic mechanisms underlying PR are still not well defined. Several authors have supported the viral pathogenetic hypothesis, identifying the involvement of HHV‐6 and ‐7 based on immunological mechanisms, which seem to be typical mechanisms against a viral infection: elevated mononuclear cells, CD4 T cells, and Langerhans cells along with a higher concentration of IFN‐α were found in the dermis and the sera, respectively, of patients with PR.^12^

However, in 1970 Burch and Rowell had already speculated about the possible autoimmune etiology of PR. Gangemi et al. studied the involvement of cytokines and chemokines in PR, in particular the role of fractalkine (CX3CL1), belonging to the δ‐chemokine family. Fractalkine is expressed on the surface of T cells and dendritic cells (above all during their maturation) and it is upregulated at the site of dermal inflammation, such as in psoriasis and the lichen planus. According to Gangemi et al., these findings suggest the main role of the immunological system and dendritic cells and cellular immunity in the pathogenesis of PR.^13^ Furthermore, fractalkine binds its receptor CX3CR1 and activates different intracellular signaling pathways, recruiting and activating CD8 and CD4 T cells, natural killer cells, and monocytes.

Another cytokine studied in the sera of PR patients is IL‐22, which promotes antimicrobial defense and is involved in dermal inflammation in some diseases, such as psoriasis. Significantly high levels of IL‐22 were found in patients affected by PR, thus supporting the viral pathogenetic hypothesis. Firstly, the increased levels of IL‐22 could be responsible for the inflammatory response, which self‐limits the spread of the viral infection and the related disease.^14^ Moreover, viruses, such as HHV, stimulate the expression of IL‐21 mRNA. IL‐21 reaches the maximum levels at the onset of the adaptive immune response, thus stimulating the differentiation and proliferation of activated leukocytes and performing an autocrine action on Th17 cells. In this way, other mediators, such as IL‐17, IL‐22, and IL‐21, are produced. Gangemi et al. stressed the viral pathogenic hypothesis and the involvement of the cellular immune response, suggesting that a viral antigenic trigger could amplify IL‐22 secretion by an autocrine mechanism, through Th17 stimulation and IL‐21 production.^14^

**FIGURE 2:**
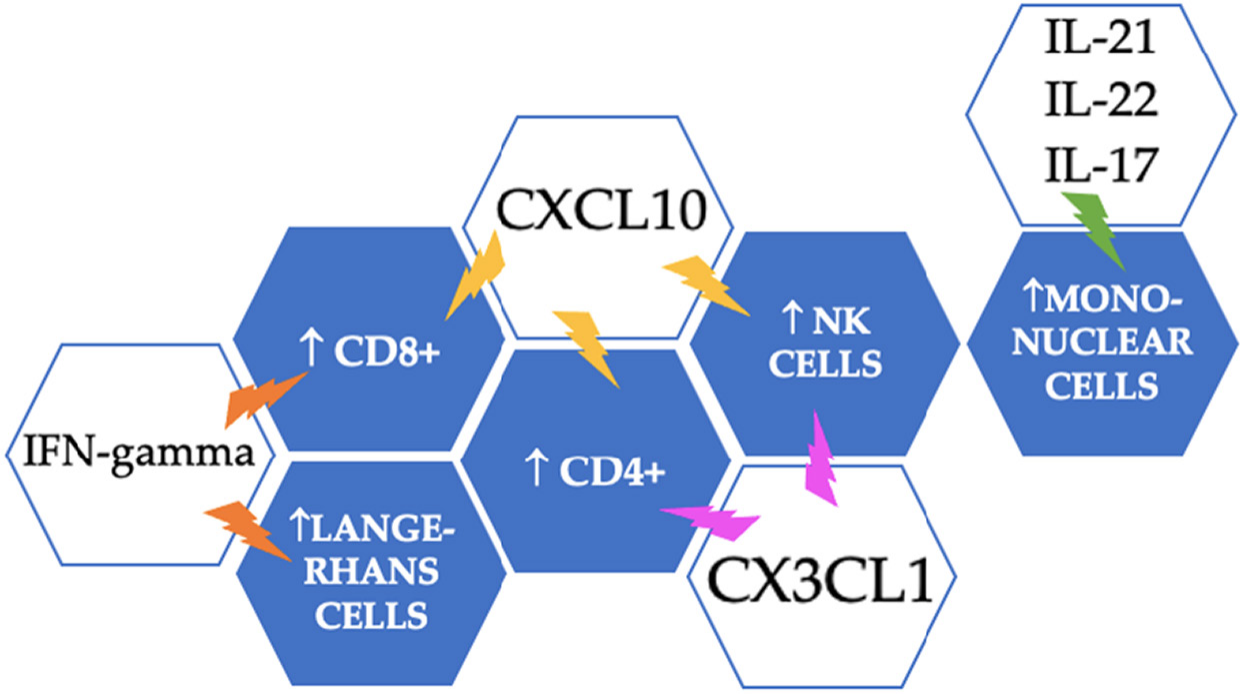
The immunological mechanisms of PR have been summarized. The markers suggesting the viral hypothesis of PR are represented in the blue boxes. In the white boxes, the mediators with elevated serum levels in patients with PR have been summarized. The hive shows through the colored lightning bolts the effects that each mediator has on cells during PR. Orange, the effects of IFN‐γ on the cells; yellow, the effects of CXCL10 on cells; purple, the action of CX3CL1 on cells; green, the increase of mononuclear cells mediated by IL‐21, IL‐22, and IL‐17. CX3CL1, C‐X3‐C motif ligand 1; CXCL10, C‐X‐C motif chemokine ligand 10; IFN‐gamma, Interferon‐gamma, IL‐17,21,22, Interleukin‐17,21,22.

Another study of the cytokines involved in the pathogenesis and course of PR was conducted by Drago et al. in 2015.^15^ Higher levels of IL‐17, IFN‐γ, vascular endothelial growth factor (VEGF), and C‐X‐C motif chemokine ligand (CXCL10) were detected. IL‐17 stimulates the release of antimicrobial peptides, pro‐inflammatory cytokines and chemokines. The increase of IL‐17 could be considered indirect evidence of the viral etiology of PR. IFN‐γ, produced by T cells and natural killer cells, promotes the cytotoxicity of virus‐specific T cells, and activates gene expression. These genes exert pro‐inflammatory effects by increasing antigen processing and presentation, and anti‐inflammatory effects due to their apoptotic and antiproliferative functions. CXCL10 or interferon‐γ‐induced protein 10 (IP‐10) is an IFN‐inducible chemokine, produced by neutrophils and keratinocytes, which recruits natural killer cells, CD4 and CD8 T lymphocytes.^15^ In Figure 2, the hive represents the effect that each mediator determines at the level of skin cellularity in patients with PR.The purpose of this review is to analyze the connection between SARS‐CoV‐2 and PR. Our aim is to investigate whether COVID‐19 can cause cutaneous manifestation compatible with PR, through an immune dysregulation or if it is caused by the reactivation of HHV‐6/HHV‐7 followed by COVID‐19 infection.

## 2 METHODS

We carried out a PubMed search including all articles in English published until November 2021. We used the keywords “pityriasis” and “COVID‐19”, without time limits.

## 3 RESULTS AND DISCUSSION

Our analysis yielded 65 articles of which 53 were not considered because the title and/or abstract suggested that they did not cover the topic of interest (*n* = 28), were not written in English (*n* = 3), or were not relevant to the association between PR and COVID‐19 (*n* = 22). The others, which reported on a possible association between PR and COVID‐19 (*n* = 12), were included. Table 1 reports cases with simultaneous infection of COVID‐19 and PR, dividing the cases into two groups: those with an initial diagnosis of COVID‐19, followed by PR, and vice versa.

**TABLE 1:** Cases with a related diagnosis of COVID‐19 and PR

Author	Article type	Country	n	Age	Sex	COVID symptoms	Time lapse with PR onset
Cases with COVID‐19 diagnosis before PR diagnosis							
Veraldi S^20^	Letter	Italy	2	26 25	F F	Mild Mild	56 days 42 days
Birlutiu V^21^	Case report	Romania	1	54	F	Mild	7 days
Öncü INS^10^	Letter	Turkey	1	10	M	Mild	10 days
Welsh E^22^	Letter	Mexico	1	49	M	Mild	7 days
Drago F^23^	Letter	Italy	1	16	M	Mild	21 days
Busto‐Leis JM^24^	Letter	Spain	2	26 48	F F	Mild Asymptomatic	30 days
Cases with PR diagnosis before COVID diagnosis							
Ehsani AH^25^	Letter	Iran	1	27	M	Moderate	3 days
Merhy R^26^	Letter	Lebanon	1	26	F	Mild	2 days
Enguix DM^18^	Letter	Spain	1	19	F	Asymptomatic	
Johansen M^19^	Letter	Georgia	2	39 23	F F	Asymptomatic Asymptomatic	
Paolino G^27^	Letter	Italy	1	33	F	Mild	25 days
Veraldi S^28^	Letter	Italy	2	26 21	M M	Mild Mild	

COVID, Coronavirus disease; PR, Pityriasis rosea.

Recent studies have reported an increase in the number of cases of PR during the SARS‐CoV‐2 pandemic. A Turkish multicenter study described the change in the profile of dermatology patients visiting outpatient clinics during the COVID‐19 outbreak. Although the number of patients decreased, the frequency of some diseases, including PR and VZV manifestations, increased significantly after the pandemic. It is interesting to note how virally transmitted diseases have increased in incidence, despite the social distancing imposed by anti‐COVID regulations. VZV has been suggested as being an indicator or complication of COVID‐19 infection.^16^ In accordance with Kartal’s study, Dursun et al. reported that the percentage of patients with PR increased during the pandemic compared to the pre‐pandemic era (from 0.8% during the pre‐pandemic era to 3.9% during the pandemic).^17^ Also, Turan et al. found a statistically significant (*p* < 0.05) increase in the frequency of dermatological diagnosis, among them PR (*p* < 0.013).^9^ Our results show how PR can be the first manifestation of COVID‐19, but at other times, conversely, COVID‐19‐related symptoms may predate the onset of the rash. Other cases highlight the need for SARS‐CoV‐2 testing in patients presenting with PR‐like eruptions, even if otherwise asymptomatic, for diagnosis and contact tracing.^18^, ^19^

Several viral infections activate innate and adaptive human immune responses. In some circumstances, as with the previous SARS‐CoV, Middle East respiratory syndrome coronavirus (MERS‐CoV), and SARS‐CoV‐2 pandemics, the monocytic–macrophage system may produce an upregulated immune response, a severe inflammatory systemic state, and damage to lungs and other internal organs.

A recent review highlights that the dysregulation produced by the cytokine storm plays a central role in other viral infections that occurred in the last century: SARS‐CoV (2002/2003), MERS‐CoV (2012), H1N1 (2009), and Spanish flu (1918–1919). All these respiratory infections are characterized by hyperinflammation, an alteration of T‐cell function, the activation of endothelial cells, and consequently, a state of hypercoagulability. Many common actors of these mechanisms have been found among the various viral infections mentioned, including IL‐1β, TNF‐α, IFN‐γ, IL‐17, monocyte chemoattractant protein 1 (MCP1), IL‐6, CXCL10, and CXCL8, which are also very much involved in the pathogenesis of SARS‐CoV‐2.^29^

The wide range of causes and the underlying mechanisms of the skin involvement in viral infections can be categorized into two groups: (i) viruses directly affecting the skin or inducing a host immune response, thus causing cutaneous manifestations; and (ii) viruses as a possible inducer of the reactivation of another virus.

### 3.1 Viruses directly affecting the skin or inducing host immune response thus causing cutaneous manifestations

Cutaneous manifestations of SARS‐CoV‐2 infections were described extensively in 2021, although they were not necessarily visible in all the patients. This could be due to the different immunological responses among different individuals or to the role of gene expression. Most patients with severe cases of COVID‐19 have high levels of pro‐inflammatory cytokines and infection‐related biomarkers. As argued by Gangemi et al., PR could be considered an immunological disease because of the involvement of a high number of chemokines and cytokines,^14^ and the contact between the immune system and an infectious pathogen could trigger the activation of immunological mechanisms.

Welsh et al. reported the first case of PR‐like dermatosis confirmed by skin biopsy. Immunohistochemistry (IHC) with SARS‐CoV/SARS‐CoV‐2 spike protein was positive in endothelial cells and perivascular lymphocytes. These findings suggest that viral infection of lymphocytes or endothelial cells by SARS‐CoV‐2 may cause these manifestations.^22^ Moreover, during the acute phase of COVID‐19 the lesions appear more erythematous and purpuric, indicating a general inflammatory state associated with the SARS‐CoV‐2 infection.^27^ Similarly, the appearance of other skin manifestations, such as erythema multiforme (EM), is linked to the involvement of lymphocytes and endothelial cells by SARS‐CoV‐2.

Erythema multiforme is mainly associated with infectious agents, above all HSV. The pathophysiological mechanism of COVID‐19‐related skin eruptions could be a hypersensitivity reaction mediated by lymphocytes targeting SARS‐CoV‐2 antigens in the skin, like what was reported for EM associated with other infections, especially HSV. In particular, CD8^+^ T lymphocytes induce the apoptosis of scattered keratinocytes and lead to satellite cell necrosis. However, in most cases, EM eruptions are not related to a severe evolution of COVID‐19.^30^

Moreover, skin biopsies have shown SARS‐CoV‐2 envelope and spike proteins, as well as striking numbers of complements in vascular endothelial cells in patients with retiform purpura. The activation of complement, therefore, plays a central role in initiating this cutaneous vascular disorder, and COVID‐19‐associated skin disorders generally.^4^

On the other hand, the autoreactive antibody generation could occur after an infection, such as SARS‐CoV‐2 infection, and could be involved in a molecular mimicry mechanism.

Indeed, Gallman et al. identified 26 autoantibodies targeting the vasculature, connective tissue, and skin. However, in the same review, they suggest that molecular mimicry is unlikely to be the only mechanism involved in self‐reactivity. In fact, “bystander activation” might be involved, in which an exaggerated immune response against a virus causes tissue damage and the exposure of self‐antigens. The latter can activate local autoreactive B and T cells, leading to autoimmunity.^4^

**FIGURE 3:**
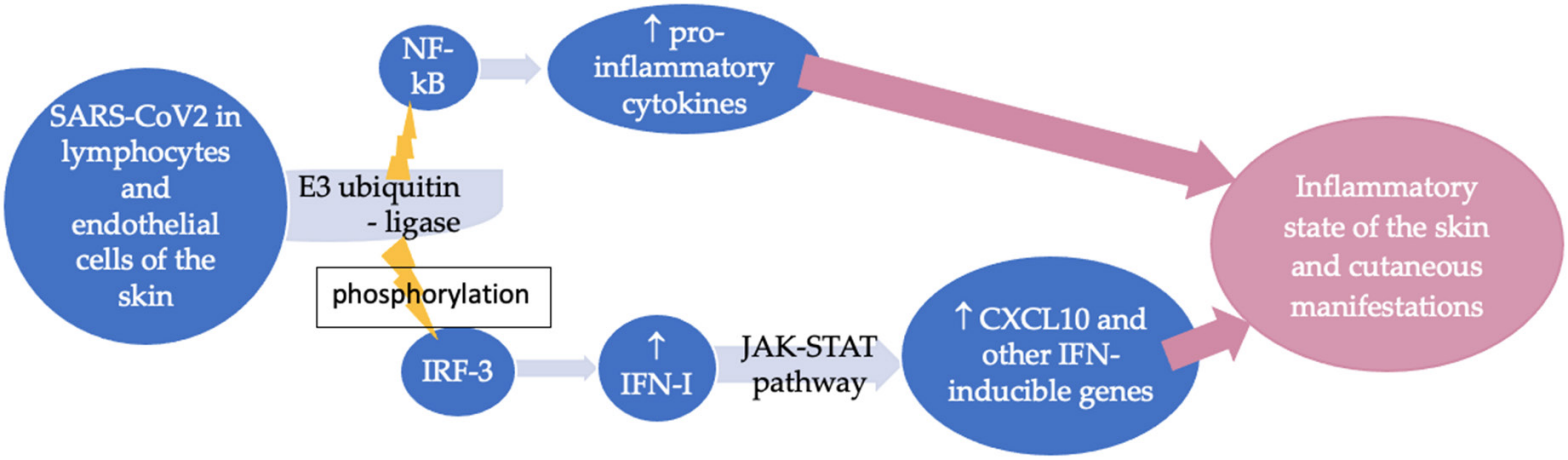
The mediators and the pathways activated by SARS‐CoV‐2 infection, which could cause a direct inflammatory state of the skin and the consequent cutaneous manifestation of PR, are summarized. CXCL10, C‐X‐C motif chemokine ligand 10; IFN‐I, Interferon‐I; IRF‐3, Interferon regulatory factor‐3; JAK, Janus kinase; NF‐kB, nuclear factor kappa‐light‐chain‐enhancer of activated B cells; STAT, signal transducer and activator of transcription.

Severe acute respiratory syndrome coronavirus 2 can affect host cells through two types of receptors: the angiotensin‐converting enzyme 2 (ACE2) and transmembrane serine protease 2 (TMPRSS2) expressed in epithelial cells and endothelial cells. The distribution of these receptors on the pericytes, that envelope the endothelial cells, could explain the features of COVID‐19‐related cutaneous manifestation. Upon entry into epithelial cells, the viral RNA proteolysis, the translation of viral proteins, and the assembly of new viruses occur. The expression of ACE2 receptor on the surface of endothelial cells facilitates SARS‐CoV‐2 entrance and promotes the activation of monocytes, T cells, and neutrophils.^31^ The involvement of cutaneous blood vessels and the release of inflammatory mediators could be responsible for skin manifestations as a direct action of the SARS‐CoV‐2, as shown in Figure 3. The viral genome, through E3 ubiquitin ligase, activates nuclear factor (NF)‐κB, which acts as a transcriptional activator for pro‐inflammatory cytokines. Moreover, the viral genome, through ubiquitin kinase, phosphorylates IFN‐regulatory factor 3 (IRF‐3), which promotes the synthesis of IFN‐I. IFN‐I, via interferon‐α/β receptor (IFNAR) expressed on the surface of cell membranes and via the Janus kinase (Jak)–signal transducer and activator of transcription (STAT) pathway, activates the expression of IFN‐inducible genes, such as CXCL10. High levels of CXCL10 were found in the sera of patients affected by PR in a study conducted by Drago et al. in 2015^15^ and in sera of SARS‐CoV2 subjects.^2^ In addition, Ocampo‐Candiani et al. suggest that the hypercytokinemia and expression of IFN‐inducible genes during SARS‐CoV‐2 infection could explain the onset of cutaneous manifestations in symptomatic patients of COVID‐19 infection, through the overexpression of genes involved in inflammation.^32^ Lastly, Gallman et al. reported an increased expression of type I IFN‐induced genes in the skin biopsies of chilblain lesions in COVID‐19 patients, and many other studies have proposed that COVID‐19‐associated chilblains and other cutaneous manifestations may be the result of an overactive type I IFN response.^4^ Murdaca et al. reported that inflammatory components during viral infections, such as SARS‐CoV and MERS, activate transcriptionally regulated genes in endothelial cells, including NF‐κB binding sites in their promoter regions, and gene analysis performed exerting mutations demonstrated the pivotal role of NF‐κB in the expression of pro‐inflammatory genes.^29^

### 3.2 Viruses as a possible inducer of the reactivation of another virus, such as HHV‐7 and VZV

To shed light on the role of human herpesvirus in PR, several studies have measured HHV‐6 and HHV‐7 through the detection of their DNA load in plasma, in peripheral blood mononuclear cells (PBMC), expression of mRNA, and specific viral antigens in PR lesions, all markers of systemic active infection.^33^, ^34^, ^35^ These studies established a causal role of systemic active HHV‐6 and/or HHV‐7 reactivation in the pathogenesis of PR. In addition, Katsafanas et al. reported that HHV‐7 operates as a primer providing the reactivation of latent HHV‐6. Once reactivated, the latent HHV‐6 genome predominates, causing HHV‐7 to disappear or preventing its detection by PCR or serology.^36^

The above‐mentioned data could confirm the causal association between PR and active HHV‐7 or, to a lesser extent, HHV‐6 infection. Therefore, just as HHV‐7 reactivates HHV‐6 leading to the cutaneous manifestations of pityriasis, in the same way, SARS‐CoV‐2 could act as a transactivator agent triggering HHV‐6/7 reactivation and indirectly causing the clinical manifestation of PR,^37^ although the concomitant involvement of other viruses has not been serologically excluded.^21^

Viral reactivation can occur through various mechanisms and molecular pathways. Many pro‐inflammatory cytokines reported in SARS‐CoV‐2 patients could promote the reactivation of other latent viruses, such as HHV‐6 and HHV‐7. SARS‐CoV‐2 infection impairs not only the number of lymphocytes, leading to lymphopenia, but also the immune response, with severe immune‐mediated injury. High concentrations of IL‐1β, CXCL10, MCP1, and IL17 have been detected in affected subjects. All of them can stimulate Th1 response. High levels of TNF‐α, MCP1, and MIP1α have been found in patients in intensive care units, suggesting a link between cytokine production and the gravity of the illness.^2^ The serum of CXCL10 or IP‐10 level was found to be significantly high in SARS‐CoV‐2 patients, and it has been associated with the severity of COVID‐19.^38^ IP‐10 was also found to be upregulated in PR patients compared to controls.^15^ CXCL10 is produced via IFN‐inducible genes and stimulates its receptor CXCR3, expressed both in circulating and memory T cells. Consequently, the CXCL10/CXCR3 axis could be involved in reactivating HHV‐6, thus leading to the pathogenesis of PR in infected subjects. This mechanism has already been observed by Yang et al. in the reactivation of HHV‐6 in drug reaction with eosinophilia and systemic symptoms (DRESS) patients. In this research the IP‐10 levels were significantly higher in the patients with DRESS with HHV‐6 than in the patients with DRESS without HHV‐6 reactivation, although these findings did not reach statistical significance. They hypothesize that the IP‐10/CXCR3 axis might drive CXCR3^+^ effector CD4^+^ and CD8^+^ T cells to inflammatory sites; thus, the HHV‐6 reactivation could be associated with a stronger CXCR3‐driven Th1‐cell response.^38^ Liu et al. argue that CXCL10 facilitates the reactivation of other viruses, such as the HSV, by stimulating virus replication in macrophages and lymphocytes.^39^

**FIGURE 4:**
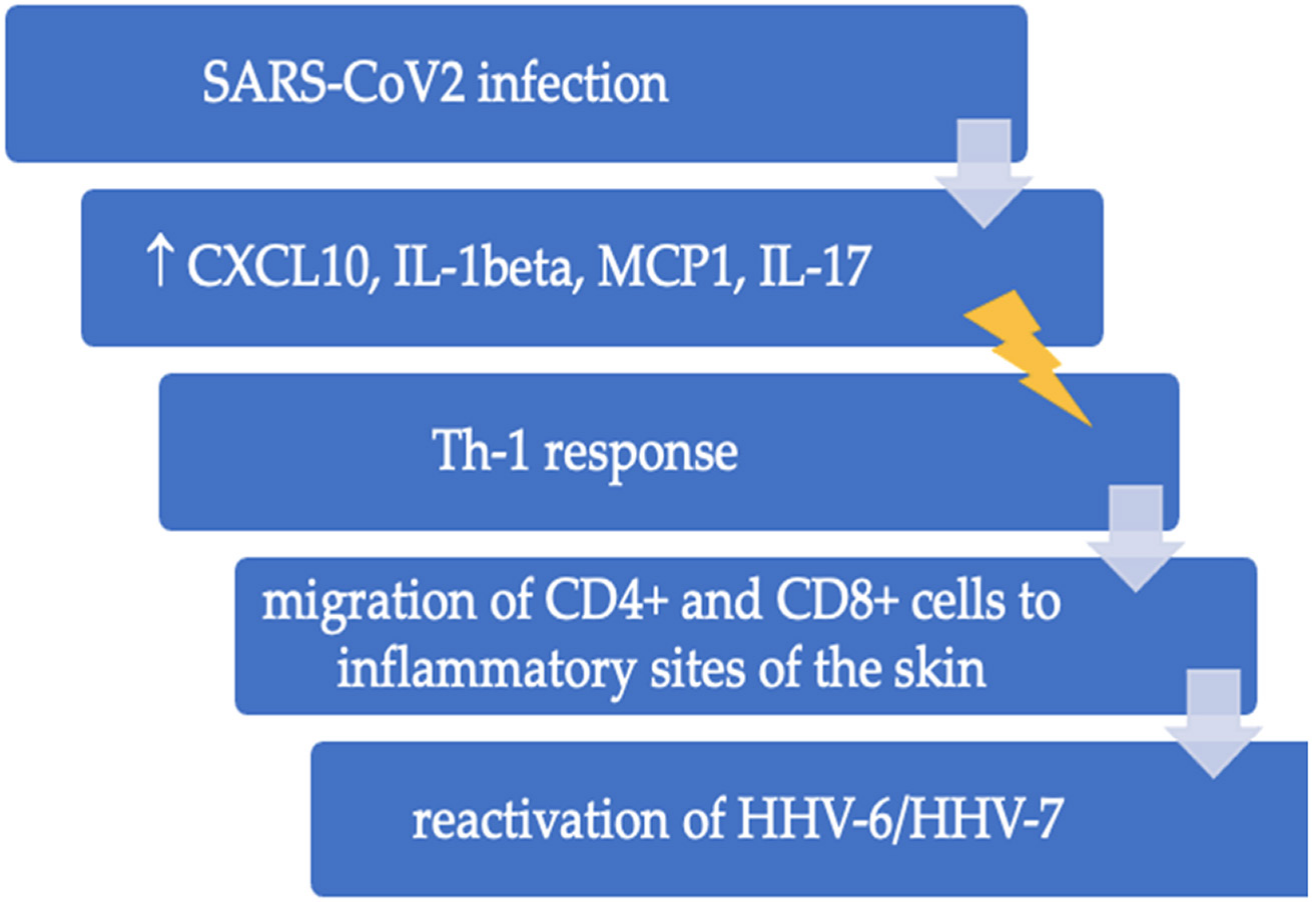
The main mediators, which could activate the Th1 response and the reactivation of HHV, are summarized. CXCL10, C‐X‐C motif chemokine ligand 10; HHV, human herpes virus; IL‐1beta,17, interleukin‐1beta,17; MCP1, Monocyte chemoattractant protein‐1.

**FIGURE 5:**
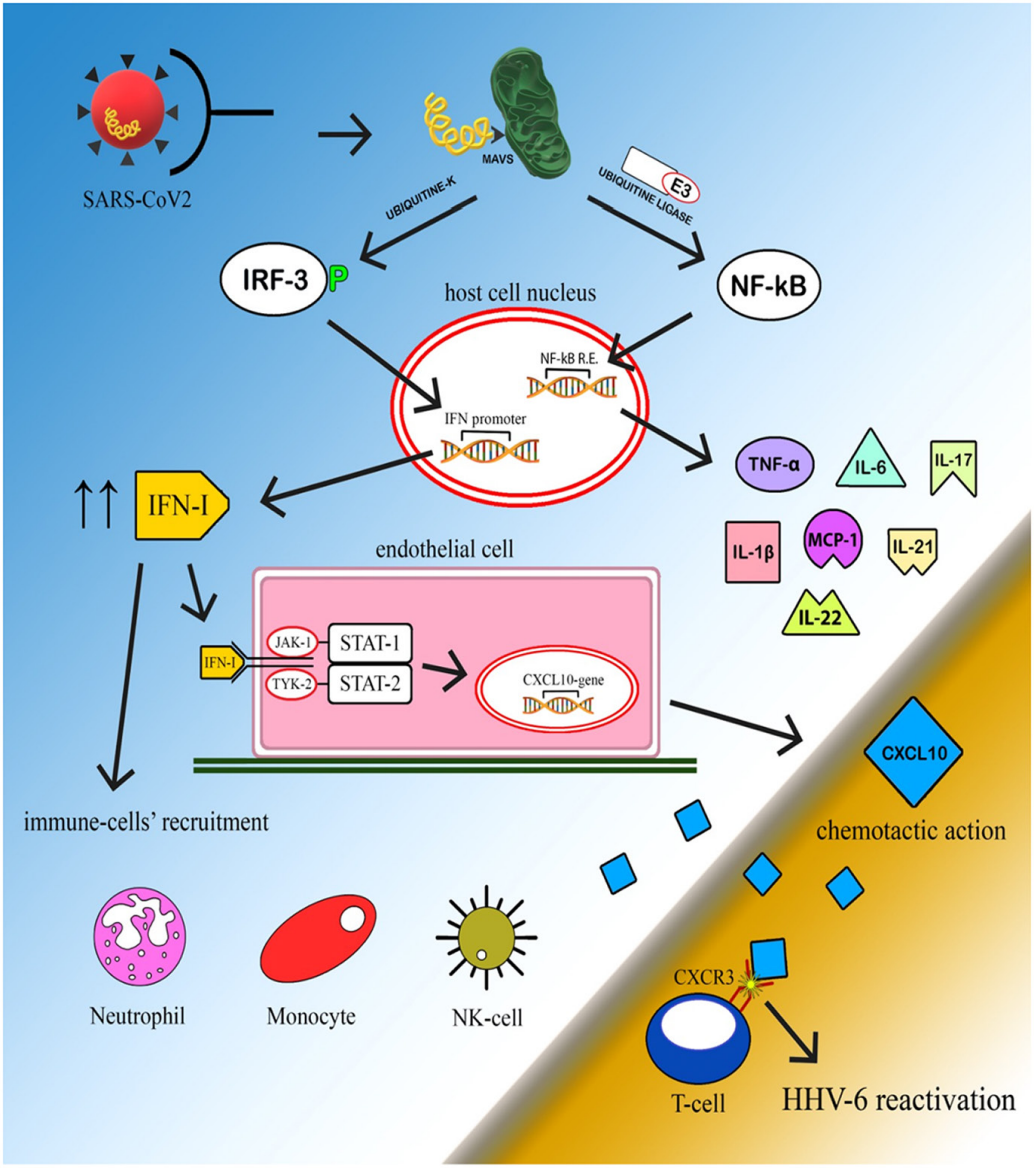
The main immunological mechanisms probably involved in the pathogenesis of PR following SARS‐CoV‐2 infection are described: a direct pathway ending with the production of pro‐inflammatory cytokines and an indirect pathway involving IFN‐inducible genes. The yellow helical structure represents viral RNA. The green structure is a mitochondrion. CXCR3, C‐X‐C motif chemokine receptor 3; CXCL10, C‐X‐C motif chemokine ligand; HHV, human herpesviruses; IL, interleukin; IFN, interferon; IRF, IFN‐regulatory factor; JAK, Janus kinase; MAVS, mitochondrial antiviral‐signaling protein; MCP‐1, monocyte chemoattractant protein 1; NF‐κB R.E., nuclear factor‐κB responsive element; NK, natural killer; “P”, phosphorylation; STAT, signal transducer and activator of transcription; TNF, tumor necrosis factor.

These results suggest that these inflammatory mediators may synergistically modulate PR pathogenesis *(*Figures 4 and 5 *)*.

Another mechanism involved in viral reactivation could be lymphopenia. As observed by Veraldi et al. in patients infected with SARS‐CoV‐2 who experience prolonged symptoms, termed “long‐haulers” or said to have “long COVID”, it is possible that lymphopenia plays a role in viral reactivation.^20^ Several studies have reported alterations in the numbers and functions of T cells in infected patients, correlating T‐cell subset alteration with an inflammatory condition.^2^ Shah et al. argued that inflammatory status is also responsible for several apoptotic genes and p53 signaling molecule upregulation, which may contribute to lymphopenia.^40^ Just as SARS‐CoV‐2 can stimulate HHV‐6 activation, in the same way it was speculated that it might reactivate other latent infections, such as VZV, by acting as a transactivator agent.^16^ This could explain the higher number of patients with PR and VZV observed by Kartal et al. during the COVID‐19 pandemic.^16^

The mechanisms of VZV reactivation are analogous to those hypothesized for HHV‐6: lymphopenia, CXCL10/CXCR3 axis, and cytokine networks. Diez‐Domingo et al. suggested that the reactivation of VZV in COVID‐19 patients could be explained through a wide range of T‐cell immune dysfunctions (lymphopenia and lymphocyte exhaustion).

Lymphopenia could be caused by the direct infection of lymphocytes by SARS‐CoV‐2 (the ACE2 receptor is expressed on the surface of lymphocytes) or by the apoptosis of lymphocytes promoted during a cytokine storm, with TNF‐α and IL‐6 playing a key role.^41^ Moreover, Saati et al. described a case of reactivation of VZV in a young immunocompetent patient with mild symptoms. They observed significantly lower T‐cell and CD8 levels, indicating that SARS‐CoV‐2 may directly infect lymphocytes, which ultimately results in the inability to perform normal antiviral functions.^42^

The role of the CXCL10/CXCR3 axis was studied by Steain et al. during experimental and natural VZV infection. They found upregulated CXCL10 expression in the dorsal root ganglion during experimental infection and, most significantly, also *in vivo* during the reactivation of naturally‐infected dorsal root ganglion. In addition, infiltrating cells expressing CXCR3, probably recruited by CXCL10, were identified in the dorsal root ganglion during VZV reactivation.^43^ Yu et al. conducted a bioinformatic analysis of COVID‐19 and VZV‐associated genes and found that STAT3 acts as a central hub in the protein–protein interaction networks of both diseases, thus suggesting that COVID‐19 might induce excessive Th17 differentiation, thereby increasing the circulating level of IL17A, which triggers VZV reactivation.^44^

Such evidence would seem to demonstrate the transactivator function of SARS‐CoV‐2 towards other viruses, such as HHV‐6/7 and VZV.

Finally, the skin manifestations resulting from anti‐SARS‐CoV‐2 vaccines are worthy of discussion. It is well known that vaccines against a specific pathogen can cause similar manifestations given by the infection itself, however of a smaller entity. An example is represented by episodes of post‐vaccination thrombosis, which are less severe and rarer than those caused by SARS‐CoV‐2 infection. In fact, in preclinical trials of the Moderna vaccine, the incidence of PR was 0.9%.^45^ However, several researches have reported cases of PR after COVID‐19 vaccines with an incidence higher than that reported by the EMA in pre‐clinical studies.^17^, ^46^ It must be considered that the vaccination campaign is still ongoing worldwide, so these data are reliable, but not definitive. This suggests that anti‐SARS‐CoV‐2 vaccines can also cause immune dysregulations resulting in a commitment of the immune system and a deviation of its cell‐mediated control over other latent viruses.^47^ The reactivation of herpes viruses after vaccine administration could be responsible for the same extrapulmonary manifestations of the infection, but to a lesser extent.

## 4 CLOSING REMARKS

In conclusion, in the literature, many cutaneous manifestations have been described in patients affected by SARS‐CoV‐2. Our analysis of the mechanisms underlying the onset of PR cutaneous manifestations, suggests two causes.

Firstly, the cytokine storm, which characterizes SARS‐CoV‐2 infection, could be responsible for a direct effect on the skin, resulting in cutaneous inflammation.^48^ In fact, during the COVID‐19 pandemic there has been an increased incidence of other cutaneous diseases, such as urticaria, psoriasis, alopecia, and chilblain‐like skin lesions.^16^ Gallman et al. believe that dysregulation of the humoral immune response is a feature of SARS‐CoV‐2 infection and may contribute to skin manifestations of COVID‐19‐associated disease, also through autoimmunity mechanisms, such as molecular mimicry or bystander activation.^4^ These cutaneous manifestations could be an indicator of latent COVID‐19 infections because these skin lesions can precede the onset of respiratory SARS‐CoV‐2 symptoms. For these reasons, IHC analysis of skin biopsies may represent a useful tool in selected patients with a suspected COVID‐19 diagnosis.^22^

The second mechanism that may be involved in the increased incidence of PR is related to the fact that the SARS‐CoV‐2 infection acts as a reactivator for other latent viruses. The likely connection between PR and the herpes virus family has been investigated in the literature, in particular HHV‐6 and HHV‐7; however, other viruses, such as VZV, have not been ruled out. The reactivation could be related to lymphopenia or lymphocyte exhaustion. However, COVID‐19 is an immunological disease and is associated with cytokine–cytokine receptor interaction, the Jak–STAT signaling pathway and the IL‐17 signaling pathway; thus, cytokines and chemokines have indirect effects, activating various pathways. Increased levels of IFN induce the activation of the CXCL10/CXCR3 axis, and this axis plays a role in reactivating HHV‐6 and VZV. The differentiation of naive CD4^+^ T cells into Th17 cells induces increased levels of IL‐21, IL‐22, and IL‐17, which are higher in patients with PR.

More studies are needed to define the connection between COVID‐19 and the pathogenesis of PR. It could be useful to measure the levels of cytokines and chemokines in patients' serum and to evaluate the various pathways in common between these two infectious and immunological diseases. As in SARS‐CoV‐2, cutaneous manifestations were also seen in SARS‐CoV, MERS‐CoV, H1N1 influenza A, and Spanish flu. All these pandemics have similar immunological mechanisms secondary to a wide spectrum of different immune dysregulation disorders with the continuous activation and expansion of immune cells, lymphocytes, and macrophages.

Such similarities should be considered in future virus‐related pandemics. In fact, skin manifestations may represent the first symptom of the disease and, therefore, the physician needs to recognize them to make a prompt diagnosis. The spread of COVID‐19 worldwide has overburdened the global health system, requiring urgent reorganizations of the routine and planning of outpatient activities. Telemedicine can act as a time‐effective substitute of face‐to‐face visit representing an excellent aid for the disposal of waiting lists, already widely exploited during the past epidemics of SARS‐CoV, MERS‐CoV, Ebola, and Zika. This health‐care procedure can guarantee continuity of care, meanwhile reducing the risk of COVID‐19 transmission, ensuring at the same time an adequate and safe triage.^49^ Free telecommunication systems like WhatsApp, email, Skype, or Zoom have been largely used. The advances in audio, visual, and data telecommunication technologies have made it easier for physicians to communicate with remotely situated patients. This is particularly relevant to dermatology as visual cues are the keystone in identifying most dermatological pathologies.^50^ The efficacy of this new system always depends on the quality of the video, images sent, and patient compliance. Nevertheless, legislation and guidelines for putting teledermatology in practice whilst protecting patient privacy and data security are needed.

We hope that the evidence given in this review will be useful in the identification, management, and treatment of future pandemic viral infections in a scenario based on precision medicine.

## CONFLICT OF INTEREST

None declared.

## References

[1] WHO Coronavirus (COVID‐19) Dashboard.

[2] Allegra A , di Gioacchino M , Tonacci A , Musolino C , Gangemi S . Immunopathology of SARS‐CoV‐2 infection: immune cells and mediators, prognostic factors, and immune‐therapeutic implications. Int J Mol Sci. 2020;21:4782. 10.3390/ijms21134782 PMC7370171 32640747

[3] Khesht AMS , Karpisheh V , Saeed BQ , Zekiy AO , Yapanto LM , Afjadi MN , et al. Different T cell related immunological profiles in COVID‐19 patients compared to healthy controls. Int Immunopharmacol. 2021;107:828. 10.1016/j.intimp.2021.107828 PMC8162824 34091116

[4] Gallman AE , Fassett MS . Cutaneous pathology of COVID‐19 as a window into immunologic mechanisms of disease. Dermatol Clin. 2021;39:533–43. 34556243 10.1016/j.det.2021.05.008 PMC8297957

[5] Do MH , Stewart CR , Harp J . Cutaneous manifestations of COVID‐19 in the inpatient setting. Dermatol Clin. 2021;39:521–32. 34556242 10.1016/j.det.2021.05.011 PMC8162718

[6] Schwartzberg LN , Advani S , Clancy DC , Lin A , Jorizzo JL . A systematic review of dermatologic manifestations among adult patients with COVID‐19 diagnosis. Skin Health Dis. 2021;1:e20. 34235511 10.1002/ski2.20 PMC8250095

[7] Saade A , Moratelli G , Azoulay E , Darmon M . Herpesvirus reactivation during severe COVID‐19 and high rate of immune defect. Infectious Diseases Now. 2021;51:676–9. 34332165 10.1016/j.idnow.2021.07.005 PMC8317452

[8] Kutlu Ö , Metin A . Relative changes in the pattern of diseases presenting in dermatology outpatient clinic in the era of the COVID‐19 pandemic. Dermatol Ther. 2020;33:e14096. 32869938 10.1111/dth.14096

[9] Turan Ç , Metin N , Utlu Z , Öner Ü , Kotan ÖS . Change of the diagnostic distribution in applicants to dermatology after covid−19 pandemic: what it whispers to us? Dermatol Ther. 2020;33:e13804. 32530503 10.1111/dth.13804 PMC7300472

[10] Öncü INS , Güler D , Gürel G , Yalçın GŞ . Pityriasis Rosea in a confirmed COVID‐19 pediatric patient. Actas Dermosifiliogr. 2021;112:864–5. 34305144 10.1016/j.adengl.2021.07.006 PMC8278835

[11] Drago F , Ciccarese G , Rebora A , Broccolo F , Parodi A . Pityriasis rosea: a comprehensive classification. Dermatology. 2016;232:431–7. 27096928 10.1159/000445375

[12] Guarneri F , Cannavo SP , Minciullo PL , Gangemi S . Pityriasis rosea of Gibert: immunological aspects. J Eur Acad Dermatol Venereol. 2015;29:21–5. 25200809 10.1111/jdv.12701

[13] Gangemi S , Cannavò SP , Guarneri F , Merendino RA , Sturniolo GC , Minciullo PL , et al. The CX3C‐chemokine fractalkine (CX3CL1) is detectable in serum of patients affected by active pityriasis rosea. J Eur Acad Dermatol Venereol. 2006;20:1366–7. 17062081 10.1111/j.1468-3083.2006.01721.x

[14] Gangemi S , Minciullo PL , Guarneri F , Cristani M , Arcoraci T , Spatari G , et al. Increased serum levels of interleukin‐22 in patients affected by pityriasis rosea. J Eur Acad Dermatol Venereol. 2009;23:858–9. 19646140 10.1111/j.1468-3083.2008.03067.x

[15] Drago F , Ciccarese G , Broccolo F , Ghio M , Contini P , Thanasi H , et al. The role of cytokines, chemokines, and growth factors in the pathogenesis of pityriasis rosea. Mediators Inflamm. 2015;2015:1–6. 10.1155/2015/438963 PMC4584227 26451078

[16] Kartal SP , Çelik G , Sendur N , Aytekin S , Serdaroğlu S , Doğan B , et al. Multicenter study evaluating the impact of COVID‐19 outbreak on dermatology outpatients in Turkey. Dermatol Ther. 2020;33:e14485. 33135831 10.1111/dth.14485

[17] Dursun R , Temiz SA . The clinics of HHV‐6 infection in COVID‐19 pandemic: pityriasis rosea and Kawasaki disease. Dermatol Ther. 2020;33:e13730. 32475003 10.1111/dth.13730 PMC7300497

[18] Enguix DM , MCS N , DTM R . Erupción tipo pitiriasis rosada de Gibert en una paciente asintomática con positividad para COVID‐19. Med Clin. 2020;155:273. 10.1016/j.medcli.2020.05.024 PMC7274607 32654832

[19] Johansen M , Chisolm SS , Aspey LD , Brahmbhatt M . Pityriasis rosea in otherwise asymptomatic confirmed COVID‐19–positive patients: a report of 2 cases. JAAD Case Rep. 2021;7:93–4. 33195784 10.1016/j.jdcr.2020.10.035 PMC7648492

[20] Veraldi S , Spigariolo CB . Pityriasis rosea and COVID‐19. J Med Virol. 2020;93:4068. 33205836 10.1002/jmv.26679 PMC7753377

[21] Birlutiu V , Birlutiu RM , Iancu GM . Pityriasis rosea Gibert triggered by SARS‐CoV‐2 infection: a case report. Medicine. 2021;100:e25352. 33832113 10.1097/MD.0000000000025352 PMC8036126

[22] Welsh E , Cardenas‐de la Garza JA , Cuellar‐Barboza A , Franco‐Marquez R , Arvizu‐Rivera RI . SARS‐CoV‐2 spike protein positivity in pityriasis rosea‐like and urticaria‐like rashes of COVID‐19. Br J Dermatol. 2021;184:1194–5. 33511657 10.1111/bjd.19833 PMC8013476

[23] Drago F , Ciccarese G , Rebora A , Parodi A . Human herpesvirus 6, 7 and Epstein Barr virus reactivation in pityriasis rosea during COVID‐19. J Med Virol. 2021;93:1850–1. 32970319 10.1002/jmv.26549 PMC7537064

[24] Busto‐Leis JM , Servera‐Negre G , Mayor‐Ibarguren A , Sendagorta‐Cudós E , Feito‐Rodríguez M , Nuño‐González A , et al. Pityriasis rosea, COVID‐19 and vaccination: new keys to understand an old acquaintance. J Eur Acad Dermatol Venereol. 2021;35:e489–91. 33899974 10.1111/jdv.17301 PMC8242646

[25] Ehsani AH , Nasimi M , Bigdelo Z . Pityriasis rosea as a cutaneous manifestation of COVID‐19 infection. J Eur Acad Dermatol Venereol. 2020;34:e436–7. 32359180 10.1111/jdv.16579 PMC7267489

[26] Merhy R , Sarkis AS , Stephan F . Pityriasis rosea as a leading manifestation of COVID‐19 infection. J Eur Acad Dermatol Venereol. 2021;35:e246–7. 33258224 10.1111/jdv.17052 PMC7753429

[27] Paolino G , di Nicola MR , Cantisani C , Mercuri SR . Pityriasis rosea infection in a COVID‐19 patient successfully treated with systemic steroid and antihistamine via telemedicine: literature update of a possible prodromal symptom of an underlying SARS‐CoV‐2 infection. Dermatol Ther. 2021;34:e14972. 33993616 10.1111/dth.14972 PMC8209955

[28] Veraldi S , Romagnuolo M , Benzecry V . Pityriasis rosea‐like eruption revealing COVID‐19. Australas J Dermatol. 2020;62:e333–4. 33216971 10.1111/ajd.13504 PMC7753512

[29] Murdaca G , Paladin F , Tonacci A , Isola S , Allegra A , Gangemi S . The potential role of cytokine storm pathway in the clinical course of viral respiratory pandemic. Biomedicines. 2021;9:1688. 34829918 10.3390/biomedicines9111688 PMC8615478

[30] Bennardo L , Nisticò SP , Dastoli S , Provenzano E , Napolitano M , Silvestri M , et al. Erythema multiforme and COVID‐19: what do we know? Medicina. 2021;57:828. 34441034 10.3390/medicina57080828 PMC8401222

[31] Ionescu M . COVID‐19 skin lesions are rarely positive at RT‐PCR test: the macrophage activation with vascular impact and SARS‐CoV‐2‐induced cytokine storm. Int J Dermatol. 2021;61:3–6. 34213786 10.1111/ijd.15749 PMC8444652

[32] Ocampo‐Candiani J , Ramos‐Cavazos CJ , Arellano‐Mendoza MI , Arenas‐Guzmán R , Beirana‐Palencia A , Salmon‐Demongin A , et al. International registry of dermatological manifestations secondary to COVID‐19 infection in 347 Hispanic patients from 25 countries. Int J Dermatol. 2021;60:956–63. 33963765 10.1111/ijd.15632 PMC8239526

[33] Broccolo F , Drago F , Careddu AM , Foglieni C , Turbino L , Cocuzza CE , et al. Additional evidence that pityriasis rosea is associated with reactivation of human herpesvirus‐6 and‐7. J Invest Dermatol. 2005;124:1234–40. 15955099 10.1111/j.0022-202X.2005.23719.x

[34] Drago F , Ranieri E , Malaguti F , Battifoglio ML , Losi E , Reborn A . Human herpesvirus 7 in patients with pityriasis rosea. Dermatology. 1997;195:374–8. 9529560 10.1159/000245991

[35] Watanabe T , Kawamura T , Aquilino EA , Blauvelt A , Jacob SE , Orenstein JM , et al. Pityriasis rosea is associated with systemic active infection with both human herpesvirus‐7 and human herpesvirus‐6. J Invest Dermatol. 2002;119:793–7. 12406322 10.1046/j.1523-1747.2002.00200.x

[36] Katsafanas GC , Schirmer EC , Wyatt LS , Frenkel N . In vitro activation of human herpesviruses 6 and 7 from latency. Proc Natl Acad Sci U S A. 1996;93:9788–92. 8790409 10.1073/pnas.93.18.9788 PMC38507

[37] Ciccarese G , Parodi A , Drago F . SARS‐CoV‐2 as possible inducer of viral reactivations. Dermatol Ther. 2020;33:e13878. 32558172 10.1111/dth.13878 PMC7323010

[38] Yang C , Cho Y , Hsieh Y , Hsu S , Chen K , Chu C . The interferon‐γ‐induced protein 10/CXCR3 axis is associated with human herpesvirus‐6 reactivation and the development of sequelae in drug reaction with eosinophilia and systemic symptoms. Br J Dermatol. 2020;183:909–19. 32037509 10.1111/bjd.18942

[39] Liu M , Guo S , Hibbert JM , Jain V , Singh N , Wilson NO , et al. CXCL10/IP‐10 in infectious diseases pathogenesis and potential therapeutic implications. Cytokine Growth Factor Rev. 2011;22:121–30. 21802343 10.1016/j.cytogfr.2011.06.001 PMC3203691

[40] Shah VK , Firmal P , Alam A , Ganguly D , Chattopadhyay S . Overview of immune response during SARS‐CoV‐2 infection: lessons from the past. Front Immunol. 2020;11:1949. 32849654 10.3389/fimmu.2020.01949 PMC7426442

[41] Diez‐Domingo J , Parikh R , Bhavsar AB , Cisneros E , McCormick N , Lecrenier N . Can COVID‐19 increase the risk of herpes zoster? a narrative review. Dermatol Ther. 2021;11:1119–26. 10.1007/s13555-021-00549-1 PMC8126597 33999370

[42] Saati A , Al‐Husayni F , Malibari AA , Bogari AA , Alharbi M . Herpes zoster co‐infection in an immunocompetent patient with COVID‐19. Cureus. 2020;12:e8998. 32670724 10.7759/cureus.8998 PMC7358933

[43] Steain M , Gowrishankar K , Rodriguez M , Slobedman B , Abendroth A . Upregulation of CXCL10 in human dorsal root ganglia during experimental and natural varicella‐zoster virus infection. J Virol. 2011;85:626–31. 20980518 10.1128/JVI.01816-10 PMC3014188

[44] Yu X , Li L , Chan MTV , WKK W . Bioinformatic analyses suggest augmented interleukin‐17 signaling as the mechanism of COVID‐19‐associated herpes zoster. Environ Sci Pollut Res Int. 2021;28:65769–75. 34322810 10.1007/s11356-021-15567-x PMC8318549

[45] Spikevax (previously COVID‐19 Vaccine Moderna): assessment report as adopted by the CHMP. 2021. Available at: https://www.ema.europa.eu/en/medicines/human/EPAR/spikevax

[46] McMahon DE , Amerson E , Rosenbach M , Lipoff JB , Moustafa D , Tyagi A , et al. Cutaneous reactions reported after Moderna and Pfizer COVID‐19 vaccination: a registry‐based study of 414 cases. J Am Acad Dermatol. 2021;85:46–55. 33838206 10.1016/j.jaad.2021.03.092 PMC8024548

[47] Català A , Muñoz‐Santos C , Galván‐Casas C , Roncero Riesco M , Revilla Nebreda D , Solá‐Truyols A , et al. Cutaneous reactions after SARS‐COV‐2 vaccination: a cross‐sectional Spanish nationwide study of 405 cases. Br J Dermatol. 2022;186:142–52. 34254291 10.1111/bjd.20639 PMC8444756

[48] Diotallevi F , Campanati A , Bianchelli T , Bobyr I , Luchetti MM , Marconi B , et al. Skin involvement in SARS‐CoV‐2 infection: case series. J Med Virol. 2020;92:2332–4. 32410241 10.1002/jmv.26012 PMC7272997

[49] Campanati A , Brisigotti V , Diotallevi F , D’Agostino GM , Paolinelli M , Radi G , et al. Active implications for dermatologists in ‘SARS‐CoV‐2 ERA’: personal experience and review of literature. J Eur Acad Dermatol Venereo. 2020;34:1626–32. 10.1111/jdv.16646 PMC7276770 32426855

[50] Ibrahim AE , Magdy M , Khalaf EM , Mostafa A , Arafa A . Teledermatology in the time of COVID‐19. Int J Clin Pract. 2021;75:e15000. 34714575 10.1111/ijcp.15000 PMC8646275

